# The Role of the Internet in Patient-Practitioner Relationships: Findings from a Qualitative Research Study

**DOI:** 10.2196/jmir.6.3.e36

**Published:** 2004-09-30

**Authors:** Angie Hart, Flis Henwood, Sally Wyatt

**Affiliations:** ^3^Department of Communication StudiesUniversity of AmsterdamThe Netherlands; ^2^School of ComputingMathematical and Information SciencesUniversity of BrightonUK; ^1^Centre for Nursing and Midwifery ResearchUniversity of BrightonUK

**Keywords:** Information literacy, patient-practitioner relations

## Abstract

**Background:**

Studies suggest that there has been an increase in the use of the Internet by patients in many Western societies. However, despite the many texts available on health and the Internet, not much is known about how much patients actually use the Internet to look up health information in their daily lives. We know little about what meaning this activity has for their experience of health and illness, and for their relationship with health-care practitioners.

**Objective:**

To explore patients' and practitioners' use of the Internet and to consider whether use of the Internet is changing relationships between patients and health-care practitioners.

**Method:**

The study used qualitative interviews and observations of patient–practitioner interaction. Our purposive sample of 47 patients (32 women and 15 men) had all had contact with the health services for information/treatment in relation to hormone replacement therapy (HRT)/menopause and Viagra/erectile dysfunction. The setting for the research was in general practitioners' surgeries, specialist clinics and patients' homes in the United Kingdom. Participants reflected a wide range of socio-economic groups, but most were white and British born, which, given the ethnic make-up of the town in which we conducted the research, was not surprising. In addition to patients, we interviewed 10 health-care practitioners (4 consultant doctors, 3 GPs, 2 specialist nurses, and a psychologist) about their own health information seeking practices (HISPs) and those of their patients.

**Results:**

Use of the Internet can increase patients' knowledge about their health conditions, although patients in our study were often too overwhelmed by the information available on the Internet to make an informed decision about their own care. Patients have a great deal of trust in their health-care practitioners. Health-care practitioners need to improve their own skills in Internet use. Hype around Internet use by patients appears to exceed the reality of Internet use.

**Conclusions:**

Our qualitative study suggests that use of the Internet is contributing to subtle changes in the relationship between health-care practitioners and their patients, rather than effecting the dramatic transformation some people envisage for it.

## Introduction

The rapid rise in the use of the Internet as a source of health information, as part of a general rise in Internet use, has been well documented [1-3]. Claims from policy sources, academic researchers, and patients themselves are that the increase in the use of the Internet for health information will result in positive shifts towards more equitable, or even patient-controlled, relationships between practitioners and patients [4-8]. Therefore, an understanding of Internet use may lead to further shifts in the models of practitioner–patient interaction that are used in the educational preparation of new practitioners [9-11]. However, some have drawn attention to the dangers of patients using the Internet for health information. For example, some raise the potential for misdiagnosis and exploitation [12-14]. Others suggest that Internet use can erode patients' faith in the authority of health-care practitioners [15-17]. In response to such concerns, health-care providers have established classificatory systems for evaluating the scientific worth of Web information [18,19].

## Methods

Over the past decade, the number of studies about the Internet has grown dramatically [20-24]. Some focus on particular Web sites, others on particular social groups' use of the Internet. Furthermore, we are now beginning to see a number of studies specifically about health information and the Internet [8,15,25]. However, these focus on specific groups of Internet users (for example, the “self-helpers”) and the practices they employ in such use [26], or on Internet use by patients under experimental conditions in computer laboratories [25]. These studies have illustrated well the potential for users to shape just what the Internet is or can be to individual users. However, they give us little idea of the overall significance of the Internet in relation to the other information media and sources these users are accessing, including health practitioners, in the course of their daily lives. In contrast, our research seeks to locate the Internet, for our particular sample, within a wider information landscape. Hence, the starting point for our research was people's own experience of finding information on a particular topic, using a “follow the user” approach. We focused broadly on participants' health information seeking practices (HISPs), including sources of information such as friends, health-care practitioners, NHS Direct, television, leaflets, etc. Because of this we are able to understand our participants' Internet use in the context of their other HISPs.

Our study of 47 patients (32 women, and 15 men) between the ages of 39 and 73, explored how far use of the Internet was changing the way in which they managed their health and their medical encounters. We received local research ethics approval for the study. The main method of enquiry was semi-structured interviews, each lasting between one and two hours. The interviews, which were conducted between November 2001 and November 2002, were undertaken by members of the project team and were tape-recorded and subsequently transcribed verbatim. They included questions about people's reasons for considering HRT or Viagra, their understanding of how these drugs work, and their perception of the advantages and disadvantages of their use. Participants were also asked about their awareness and use of alternative treatments. In addition, they were asked about whether and how they looked for health information generally, as well as for HRT, Viagra, and other treatments for their symptoms related to menopause or erectile dysfunction. People were asked where they look and where they find information, by what means they find it, and how they interpret and make sense of it both for themselves and in negotiation with others, including in consultation with health-care practitioners. If people used the Internet, they were asked for how long they had done so and what they used it for. If they used it for finding health information, they were asked how they did this, as well as about the advantages and disadvantages of the Internet as a source of information. Nearly half of the participants, 16 women and 5 men, were interviewed a second time, 6 to 9 months after the first interview, in order to discuss any changes in health, treatment, and information-seeking behaviour.

Descriptive statistics were generated through the use of Excel. Qualitative data were analysed using NVivo software. All the researchers were involved in coding the transcripts, and we jointly agreed the coding frame. During the initial stages of analysis we compared our transcription analyses in order to enhance the reliability of our coding.

Our sample included both Internet users and non-users. Of the 47 patients interviewed, 24 had access to the Internet: 19 of these 24 were women, and 5 were men. All participants were interviewed at least once, with a sub-section of 21 patients being interviewed at least twice (5 men and 16 women). Most interviews took place within participants' own homes, although some were conducted in offices located within health-care settings. We also observed 16 consultations between patients and health practitioners. Participants were recruited through a GP surgery and two specialist clinics (gynaecology and erectile dysfunction clinics).

We sought out patients who had had contact with the health service as a result of needing to know about two specific, but quite common, drugs/forms of treatment. For men, we chose Viagra in relation to erectile dysfunction, and for women, HRT in relation to menopause. All participants were interviewed about their HISPs in relation to their general health, and to these specific issues. Given the size and characteristics of our sample, we should point out that it may not be typical of the HISPs of patients with other health conditions. There is some evidence to suggest that patients with rare conditions are more active on the Internet [27]. Participants reflected a wide range of socio-economic groups, but most were white and British born, which, given the ethnic make-up of the town in which we conducted the research, was not surprising. In addition to patients, we interviewed 10 health-care practitioners (4 consultant doctors, 3 GPs, 2 specialist nurses, and a psychologist) about their own HISPs and those of their patients.

Of the 32 women interviewed, the average age was 55, with the youngest being 39 and the oldest 73. Eighteen were in relationships. The men were older, ranging from 54 to 81, with an average age of 66. Ten were in relationships at the time of the study. Our sample included people from a range of socio-economic groups, with varied educational experience and qualifications.

This overview of the study and the participants provides some clues as to the everyday life experiences of these people as they try to live with and inform themselves about different aspects of aging.

## Results

### Sources of Health Information

Amongst both the male and female participants in this study, medical situations were often complex. The range of symptoms, the prescribed treatments, and the after-effects experienced all varied. The possible sources of information were enormous. So, how did our participants inform themselves about health matters? All drew more or less actively on a range of sources. For both men and women, the family doctor was the most important source, and we explore this in more detail below. Family members, usually women, were the second most frequently cited source, with friends, pharmacists, and alternative practitioners also mentioned. The media used include magazines, television, World Wide Web, self-help books, newspapers, and other items such as leaflets from pharmacists or those provided by pharmaceutical companies with drugs. The most striking difference between the women and the men was that women had much more diffuse social networks, including family, friends, neighbors, and colleagues, which they drew upon to talk about their health, whereas men talked primarily with their doctors and sexual partners.

Of the 15 men in our study, 9 had access to the Internet, but only 3 used it to access health information. Of the 32 women, 24 had access to the Internet, but only 18 of them used it for this purpose. However, as we shall see below, the 21 participants who did use the Internet to look up health information did not find the experience trouble-free.

### IT Literacy

Our study showed that most participants, both patients and practitioners, were not very IT literate when it came to looking up health information on the Web. Becoming informed involves skills and competencies that relate both to the information itself and to the medium used to access that information. Amongst our participants we found many who had very few information literacy skills and others who lacked general computer literacy skills and/or Web-searching skills. Most of those who wanted to access information from the Web relied on intermediaries, and we report on this elsewhere [28]. Interestingly, one of the most damning views on self-competence came from a practitioner, a specialist nurse, rather than from a patient: “I'm not very good at it. Somebody says ‘Internet' and I think [draws in breath]. I get lost on it. That's why it terrifies me” (specialist nurse, no.2).

Some patients were aware of their lack of search skills, while others seemed unaware of, and largely unconcerned about, their rationale for accessing information the way they did. One patient participant (female participant, no. 8), for example, showed little awareness of the sources of information (publisher, organization, etc.) she finds on the Web, and expressed no interest in issues of information validity or quality, tending to trust whatever she finds there, regardless of source. While this participant was our least information-literate Internet user, many other patients were similarly uninterested in information source and validity issues, displaying low levels of information literacy. Practitioners, on the other hand, were more aware of their own skill limitations, although many were inclined not to do anything about this. Time constraints and the lack of convenient Internet access were cited as major reasons for this.

No patients reported having been given information about Internet sites from practitioners. Of the practitioners we interviewed, only one actively encouraged patients to look up information in this way, although three said that in the past they had given out such information. In our observations of consultations, we saw no examples of information about Internet sites being given out. However, some practitioners we spoke to saw encouraging Internet information seeking as a potentially useful development of their role in the future. One nurse commented that she would like to see patient Internet access in her clinic. Others mentioned that they might provide Internet addresses in their clinic. Their own lack of IT skills, and perceived lack of time, probably had a hand in such developments being slow to get off the ground.

### Patients' Trust in Practitioners 

Our research confirms the view that that despite the negative publicity health practitioners have received of late in the UK (for example, the Shipman case, in which a GP murdered many of his elderly patients by deliberately giving them the wrong medication), trust in them remains very high. Most patient participants mentioned that they would go to a known health-care practitioner first to discuss a health issue, rather than use any other source, including those to be found on the Internet. Box 1 illustrates comments made by patient participants about the trust they have in health practitioners.

**Patients' trust in practitioners**

male participant, no. 17 You can do so much on the net, you can do so much on the phone, but it is eye-to-eye contact [with a health practitioner] that counts.

female participant, no. 29 Well, I have always trusted the doctor but then of course I grew up in the era, as I'm 60, I grew up when you did trust the doctor.

female participant, no. 14 I do trust dispensers, chemists, doctors. The medical profession. Basically professionals. That's where my basic trust is.

Very few patients expressed views to the contrary; some of those who did mentioned friends and family as primary sources of health information. A minority expressed a sense of having been let down by health practitioners; however, being let down by one practitioner did not generally mean that patients developed a more diffuse sense of distrust.

Negative comments about health practitioners as information providers were rare in our research. As a result of this high level of trust, many patients did not feel the need to access alternative, or even complementary, sources of information such as those on the Internet.

### The Symbolic Power of the Internet

Despite the strong sense of trust in practitioners as a main source of health information, and despite the low levels of IT literacy in our sample, it was striking to note that many patients reified the power of the Internet, for good or for ill. We refer to this as the Internet's symbolic power, and to some extent it applied also to the practitioners. There was a strong sense amongst many participants, even those who had never used the Internet, that they should be doing so, and that they were missing out in a profound sense if they were not. In some cases, both patient and practitioner participants were clearly embarrassed by the fact that they were not “Internet savvy.” A number of participants had quite high expectations of what they would find there if only they acquired sufficient expertise. However, cases where the symbolic power of the Internet was implicitly referred to were rarely backed up by reference to actual experiences with it. Thinking specifically about the experiences of patients who reified the Internet, the quotes in Box 2 demonstrate a sense of needing to be online to reap the benefits of cheap deals, and email communication, rather than specifically to access health information. This was particularly the case for male participants. For example, one participant had used the Internet extensively to search for holidays, but when we asked him about looking up health information on the Internet, he said that he did not have time for that.

**Positive patients**

male participant, no. 11 I had heard of people looking things up on the Internet and finding out things. I thought I ought to be able to do that and I should try that.

male participant, no. 5 I want to be on the Internet, I'm missing out on a lot.

male participant, no. 17 [The Internet is] a marvellous medium and you just want to learn more and more about it. … You can get there instantly and if it doesn't give you exactly what you want there's usually a way of finding out more. …I think the Internet is marvellous…

female participant, no. 29 I haven't got it [the Internet] yet, but I've started this week a course on computers, to get to grips with the Internet and the email and buy one… you've really got to have one.

For health practitioners, the symbolic role of the Internet specifically as a source of health information was more marked. Some felt that it was an incredibly useful source of information that they, and in some cases also their patients, should be accessing. For example, a specialist nurse with little Internet experience was enthusiastic: “…we'd love it [Internet access for patients in the clinic], absolutely.”

However, it was far more common in our study for health practitioners to view the Internet as having profoundly negative powers. A few expressed their concern that Internet use would encourage patients to challenge their medical authority. Many were worried about inappropriate self-diagnosis, and about patients' taking advice from sites that did not concur with medical opinion. The Internet's role in feeding the anxieties of patients with hypochondria was raised by three practitioners, and problems with “all sorts of odd Web sites,” and patients coming in armed with printouts were mentioned by a further two. One consultant was concerned that patients would act on individualised accounts from others who post their experiences on the Web.

For the most part, these anxieties were expressed in the context of a fairly balanced view of the Internet's threats and promises. However, this was not always the case: “I am sure people are ferociously searching the Internet for information,” remarked one health-care practitioner who clearly did not approve of this (HCP, no. 1). The participant went on, “The Internet … you find yourself having to substantiate some really difficult scenarios where somebody has come armed with this information: you're on your back foot and you just don't know where to go. Can't argue about it, you are only a [HCP] and you haven't got the arguments against their specific topic which they find particularly interesting. And you are at a loss: it puts you on your back foot and makes you feel quite stupid.” However, when we asked about how often people had actually come to this person with Internet information, “only three times” was the answer.

Patients reported that some health practitioners sought to assert their authority by dismissing the patient's acquired knowledge. For example, one woman said some health practitioners had made it clear that they thought she should not look things up for herself. She felt that the view was, “you're here with me now and I'm telling you this” (female participant, no. 29).

Consultations between practitioners and patients are inter-subjective experiences in that there are always at least two people involved. As such, psychodynamic factors, as well as professionally driven agendas, are at play. Consultant psychiatrist Jeremy Holmes suggests that “perhaps rather than being motivated by altruism and scientific integrity, we are merely using our patients to bolster our fragile sense of competence and health” [29]. This perspective can be linked to debates about the limits of professional knowledge and authority, and about ways in which practitioners emotionally protect themselves from their patients, both of which go back a long way [30-32]. The health-care practitioner we discussed above was a self-described beginner in Internet use. How much, then, were practitioners' concerns about the negative power of the Internet a reflection of their own insecurities in its use, and in their own medical competence? It did seem to us that IT literacy (in terms of sorting through Web sites and evaluating the reliability of information) was as much an issue for the health-care practitioners in our study as it was for patients.

This point may have wider application in our study and beyond, although we are cautious about this since we interviewed only 10 practitioners. Nevertheless, they came from different professions and IT literacy skills were an issue for most of them, as they are for many NHS professionals [33]. White and Stancombe's discourse analysis of encounters between patients and practitioners shows that medical decision-making in the moment is a complex combination of science, art, moral action, and psychodynamic process [34]. They argue for analysis of clinical decision-making to be made on what they describe as a “re-embodied” clinician. “Putting the mind back into a feeling body—that gets angry, has friends, enemies, loyalties, vendettas, has a past and an anticipated future, becomes weary or bored—forces us to consider how we may understand the processes of judgment and intuition more adequately” [34]. The use of the Internet in health needs to be understood in this light too, and not solely in relation to debates about information quality from largely biomedical understandings.

## Discussion

To what extent, then, can our findings contribute to the debate about the changing relationships between patients and practitioners? First, our study reveals only a handful of patient participants actively challenge medical authority using the information they acquire on the Internet. Most patients articulated high levels of trust in health practitioners. Even those few who did look up health information on the Internet prior to their consultation, usually did not tell the practitioner they had done so. One way of understanding such covert practice is to see it, as Scott suggests, as “a weapon of the weak” [35] in a context where one party (the patient) significantly lacks the power to determine the actions of another (the practitioner).

Our study revealed very few examples of patients having acquired information from the Internet that actually resulted in an explicitly patient-controlled outcome. There are a number of potential reasons for this. Clearly, some practitioners were defensive about their own Internet competencies. As a result, they asserted their medical authority all the more, thereby dismissing the positive potential of the Internet, particularly if the information from it came via a patient. In other cases, and in corroboration of other studies [36,37], time limitations constrained the possibility of engaging in dialogue that might have led to a patient-controlled, or even a patient-centred, outcome. This was something that many participants in our study, both patients and practitioners, were aware of.

In their exploratory paper, Gerber and Eiser present a broad typology of how patient–physician relationships might fare in the Internet age [38]. What does our research suggest about the future of patient–practitioner relationships in the UK? If practitioners with poor IT skills do not improve their own IT literacy, use of the Internet by their patients may result in such practitioners defensively asserting their “expert opinion” all the more in the heated moment of the consultation. Relationships between patients and practitioners who are more Internet savvy can go in one of three ways. First, as we have seen, time constraints on the consultation (which studies have shown patients generally understand and respect), can lead to curtailment of opportunities for patients to become better informed. In this case, consultations are unlikely to move towards the patient-controlled end of a continuum. Rather, patients can be quickly and authoritatively steered towards the course of action preferred by the practitioner without any discussion of alternatives, even though the practitioner, and indeed the patient, might know of them.

A slightly different take on this first scenario presents us with the second one. This would involve practitioners using their technical skills to guide trusting patients to “approved sites,” information from which would reinforce the course of action favored by the practitioner—the “Internet prescription,” as Gerber and Eiser put it [38]. One doctor in our study reported steering patients' decision-making in this way. If it were to happen more widely, some may see this as Internet prescribing: information for compliance, rather than choice. However, the degree of trust patients in our study wanted to put in their practitioners potentially tempers this criticism. Clearly, some passive patients are content to be so.

The third scenario presents a view that moves more toward patient-controlled encounters. Here the privileging of practitioners' biomedical perspectives is not automatic. The perspective of one doctor in our study captures this. Thinking about the role of the Internet in relation to his dynamic with patients he suggested, “It's something about our role changing and it's something about our role becoming the processors of information rather than the providers of information” (HCP, no. 2). Other studies of HISPs suggest that the realization of this scenario is unlikely to be just around the corner for most patient–practitioner encounters [37,38]. Nevertheless, this doctor's view presents a challenge to traditional constructions of patient–practitioner relationships, and is firmly in keeping with policy shifts and the vision of the central role of patients and citizens in NHS (National Health Service) provision [40]. This doctor's view also reflects a popular discourse in the literature on health and the Internet: that relationships will be transformed [4-8,41]. Of course, the Internet is not the only mediator of information that may precipitate such a role transition from HCP-centred to patient-controlled consultations. Its symbolic importance, in drawing attention to the patient–practitioner relationship and throwing the issues of authority and trust into sharp relief, as we have explored above, is clear.

Despite the many texts available on health and the Internet, much is still unknown about how much patients actually use the Internet to look up health information in their daily lives, and what meaning this activity has for their experience of health and illness, and for their relationships with health-care practitioners [42]. Ours was a small-scale study and cannot be generalizable. At the very least though, it provides some evidence of the symbolic role of the Internet. Though slow to change, many patients and practitioners feel that they ought to be getting online. Also, whatever the future of relationships between patients and practitioners, our study demonstrates empirically, at least in one UK context, that Internet-mediated changes in their dynamics are discernible, if not dramatically so.
